# Value in care: The contribution of supportive care to value‐based lung cancer services—A qualitative semistructured interview study

**DOI:** 10.1111/hex.14089

**Published:** 2024-05-20

**Authors:** Holly Chung, Amelia Hyatt, Kate Webber, Suzanne Kosmider, Meinir Krishnasamy

**Affiliations:** ^1^ Academic Nursing Unit Peter MacCallum Cancer Centre Parkville Victoria Australia; ^2^ Department of Health Services Research Peter MacCallum Cancer Centre Melbourne Victoria Australia; ^3^ Sir Peter MacCallum Department of Oncology University of Melbourne Melbourne Victoria Australia; ^4^ Department of Nursing University of Melbourne Melbourne Victoria Australia; ^5^ Oncology Department Monash Health Clayton Victoria Australia; ^6^ Faculty of Medicine, Nursing and Health Sciences, School of Clinical Sciences Monash University Clayton Victoria Australia; ^7^ Cancer Services, Sunshine Hospital Western Health St Albans Victoria Australia; ^8^ Victorian Comprehensive Cancer Centre Alliance Melbourne Victoria Australia

**Keywords:** cancer supportive care, lung cancer, patient perspective, qualitative, value‐based care

## Abstract

**Introduction:**

Despite significant advances in the management of lung cancer, patients continue to experience a high burden of unmet need impacting quality of life and outcomes of care. Achieving value‐based health care, where investment is targeted to services that deliver optimal experience and outcomes of care relative to the cost of delivering that care, requires attention to what people value most in meeting their needs. To date there has been little attention to what matters most to patients with lung cancer (i.e., what they value) as a component of achieving value‐based cancer care. This qualitative study was undertaken to investigate components of care valued by people with lung cancer in Australia.

**Methods:**

This qualitative study used semistructured interviews with 23 people with lung cancer. Participants were recruited using a purposive sampling strategy from two metropolitan tertiary public health services. Data collected included demographic characteristics and patient perspectives regarding their priority concerns and components of care identified as most valuable in meeting their needs. Demographic characteristics of participants were analysed descriptively, and qualitative data were analysed thematically using Interpretive Description.

**Results:**

Data analysis generated three key themes: valued components of care; benefits of receiving valued care components and consequences of missed opportunities for care. The components of care valued by patients reflect the core dimensions of cancer supportive care, with particular emphasis on ongoing opportunities for consultation (screening for unmet needs) and provision of person‐centred information. The facilitation of trust between patients and their treating team, as a consequence of having these valued components evident in their care, was identified as a key characteristic of value‐based care.

**Conclusions:**

This study has identified valued components of care described by people with lung cancer. Importantly, the care components identified have been proven to improve access to and coordination of care, and demonstrate the importance of integrating supportive care into care provision to achieve value‐based cancer care.

**Patient or Public Contribution:**

This study was informed by perspectives of lung cancer patients who participated in semistructured interviews. We acknowledge that this contribution does not meet the criteria for patient and public involvement in research as defined by *Health Expectations*, but this study forms part of a larger program of cancer supportive care work being undertaken by this team, where comprehensive consumer engagement and co‐design approaches are embedded in our work.

## 1 INTRODUCTION

Lung cancer is the second most commonly diagnosed cancer and the leading cause of cancer‐related deaths worldwide.^1^ Despite substantial advances in treatment over the past two decades, improvement in survival has been modest, with just 22% of people with non‐small cell lung cancer (NSCLC) surviving 5 years from diagnosis, and 7% for people with small cell lung cancer.^2^, ^3^

Often diagnosed with advanced disease, people with lung cancer experience considerable disease and treatment‐related side‐effects that impact experience of care, quality of life and health outcomes.^4^ Breathlessness, pain, fatigue, weight loss and psychological distress have long been identified as prevalent problems reported by patients with lung cancer.^4^, ^5^ Recent data also show that patients with lung cancer are at higher risk of financial toxicity than other cancer groups,^4^, ^6^ likely exacerbated by a strong correlation between lung cancer incidence and socioeconomic disadvantage, and social determinants of health (such as smoking and education status) that impact access and outcomes of care.^7^, ^8^ Furthermore, lung cancer is one of the most‐costly cancers to treat (particularly advanced disease),^9^ imposing considerable economic burden on the health system.^10^

Importantly, the provision of timely and tailored supportive care (multidisciplinary service provision that helps people with the biopsychosocial impacts of a cancer diagnosis and its treatments),^11^ has been shown to generate substantial patient and health system benefits in a recent study of patients with NSCLC.^12^ A value‐based approach to the integration of cancer supportive care is designed to tailor the provision of supportive services, maximising health outcomes and efficiency of resource utilisation. In this context, value is defined as an approach to health system planning and delivery that aims to generate the most benefit for patients, relative to the cost of delivering that care.^13^, ^14^ Therefore, value‐based services are defined as those that are most efficient in meeting patient needs; improving experiences and outcomes that ‘matter most to me’.^15^

However, to date there has been little attention to the aspects of care that patients with lung cancer value, despite awareness of the considerable burden of unmet needs experienced by this patient population.^16^ This gap in knowledge about valued aspects of care hampers efforts to integrate these components intentionally into day‐to‐day service delivery, and thus achieve value‐based cancer care.

The aim of this study was to identify what components of care (e.g., information, social support, functional or physical care) people with lung cancer value in addressing their needs. Here, ‘value’ relates to what participants described as the services or resources that were most important and helpful to address the demands of lung cancer and its treatment. Qualitative interviews were undertaken with people with lung cancer to explore these perspectives and to understand the benefits or consequences of receiving or missing valued aspects of care. From these findings, we make recommendations for integration of the components of cancer supportive care valued by participants, as a critical enabler of achieving value‐based care for people with lung cancer.

## 2 MATERIALS AND METHODS

### 2.1 Study design

This qualitative study used semistructured interviews, grounded in a descriptive interpretivist paradigm. An interpretivist approach was chosen as it facilitates the identification of patterns and themes of experiences while acknowledging inherent variation between individual experiences.^17^ Crucially, this methodology has been developed to elicit findings relevant for clinical practice.^17^

Following ethics approval by institutional Human Research Ethics Committee (HREC) (multisite approval number: HREC/66771/PMCC), participants were recruited from two metropolitan tertiary health services (hereafter health services A and B). Data collection took place between 16 June 2021 and 13 August 2021. This paper reports on a subset of data collected as part of a larger study,^12^ for which scoping reviews of lung cancer supportive care were undertaken to inform the interview guide. Desktop audits for supportive care policy and interviews with health professionals involved in the delivery of lung cancer care at health were conducted at each site. While these data were not explicitly referred to for the purposes of this study, they form collateral data that enriched the research team's understanding of the care context from which participants reported their experiences and provided additional context during the analytical process.

### 2.2 Setting and participants

Patients were eligible to participate if they had a recently confirmed lung cancer diagnosis (<2 years and >3 months) before recruitment and had adequate English‐speaking proficiency to take part in a qualitative interview. A purposive sampling strategy was used to ensure the inclusion of a demographically diverse range of people, specifically focussing on diversity of age, identified gender and cultural background. Characteristics of both recruited and eligible participants across the two study sites were regularly reviewed during the recruitment process, and target requirements adjusted to ensure representation across different characteristics. Eligible patients attending lung clinics at each health service were identified from clinic lists. They were approached via phone by a research assistant (RA) based at each health service who explained the study aims and requirements to them. Interested individuals were sent a Participant Information and Consent Form (PICF) by email or post, depending on preference, and contacted by the RA to confirm their interest in participating. Informed consent was confirmed through signed PICFs, and verbal confirmation of consent was audio‐recorded at the start of each interview.

### 2.3 Data collection

H. C. conducted all participant interviews. The semistructured interview schedule (Table 1) was developed following a comprehensive review of published literature, discussion among the project team, and feedback from clinical and consumer experts. It was developed to explore supportive care services prioritised or valued by participants, access to and uptake of services, services that would have been accessed had they been available, and any other associated topics relevant to the focus of the study, that arose during interviews. Interviews did not explicitly ask patients to talk about valued aspects of care or things that mattered most to them, as this may have limited or curtailed description of diverse aspects of care. Rather the intent was to allow people to talk broadly about care received (or not) and how the presence of absence of a service or interaction impacted their experience. All participant interactions were telephone‐based due to restrictions relating to the COVID‐19 pandemic and all interviews were audio‐recorded.

**Table 1:** Summary of interview schedule.

Questions	Prompts
Has anyone ever explained to you all of the services which are available to you in the hospital?	This can include nonhealth related things, such as help with money, transport or family issues
Can you tell me about the services you have accessed or used in the hospital so far?	Why were you referred to these services? Have they been helpful? (why/why not, in what way) What impact do you think accessing these services had? (what have they allowed the patient to do—e.g., return to work, be able to play with the grandkids) From the help that you received, is there one service that stand out to you the most in terms of value? (how/why did it have the impact/value that it did?) Did you ever want to access services but had trouble doing so? E.g., couldn't get a referral, couldn't afford them, or another reason?
Are there any things that you need or needed help with that weren't addressed by your treating team (i.e., the Drs/nurses and allied health staff)?	This can include nonhealth related things, such as help with money, transport or family issues
If you needed help with something and it wasn't available in the hospital or offered by the hospital team, did you look outside of the hospital for help?	For example, your GP, the Cancer Council Victoria, the Lung Foundation, Internet websites, Internet peer groups (on forums, Facebook etc.)
Have you or are you experiencing any symptoms or side‐effects from your treatment?	Did you have help with managing these? At the hospital or somewhere else? Do these side‐effects impact your ability to live your life the way you prefer, e.g., can you still work, see family or friends?
Have you had any direct or ‘out of pocket costs’ from your cancer or treatment?	Have you had any indirect costs to you or your family as the result of having cancer? E.g., medications and dressings, transport costs (public transport tickets, car parking and fuel, overnight accommodation, lost work, needing to hire assistance for the home)
Aside from treating your cancer, what other things would you like your treatment or healthcare services allow you to achieve?	E.g., return to work, care for family, participate in social activities What would you need to help you achieve these things?(e.g., information to help self‐manage, additional allied health services, assistance with ADL/finances)

Abbreviations: ADL, activities of daily living; GP, general practitioner.

Demographic and clinical information including age, sex, first language, marital and employment status, highest level of education completed, Aboriginal or Torres Strait Islander origin, time since diagnosis and type/stage of cancer (if known) was collected at the start of each interview by H. C., and entered directly into a study‐specific template using REDcap electronic data capture tools.^18^

### 2.4 Project team

H. C. is a trained qualitative researcher with a background in public health, A. H., a senior qualitative researcher, provided mentorship of data collection and analysis. M. K., a senior clinician and professor in cancer nursing, provided supervision and guidance of all study aspects. S. K. and K. W. are consultant medical oncologists at the participating health services and facilitated the research as site‐specific principal investigators.

### 2.5 Data analysis

Demographic data were exported to Excel 365 and analysed descriptively (counts, proportions, mean and standard deviation as appropriate) for each site as well as aggregate.

Semistructured interviews were audio‐recorded, transcribed verbatim and deidentified, with transcripts imported into NVivo 12 (QSR International) and analysed thematically using Interpretive Description.^17^ H. C. coded all transcripts, and A. H. co‐coded two transcripts to establish trustworthiness in the coding frame.^17^ M. K. was appointed a role of third‐reviewer to resolve any discrepancies in the coding frame. Our analytic approach to these data began with our research questions which were used to inform the initial coding frame, as per the Interpretive Description methodology.^17^ Transcripts were reviewed and rereviewed against this ‘broad base’ coding frame which focussed on: the services participants found helpful, had accessed or wanted to access, in conjunction with the value (social benefits and money saved) associated with accessing these services, and the costs (social consequences and money lost) incurred when patients were not able to access these services. Then, an open coding approach was used by H. C. to identify and code distinct concepts, experiences, and perceptions by reviewing, rereviewing and comparison of transcripts to ‘make sense of patterns’.^17^ Iterations of the coding frame were cross‐checked by A. H. to ensure consistency. These open codes where then organised into hierarchical structures to explore relationships (transforming patterns into findings), and the coding frame was presented to A. H. and M. K., and interpretation discussed.^17^ This allowed drawing together the insights of H. C. who was ‘close’ to the data through data collection, cleaning and analysis with other study members who had considerable methodological (A. H., M. K.) and clinical (M. K.) expertise. There were several iterations of this step. Finally, the coded data were synthesised into narrative form that allowed the research questions to be addressed. In addition, the collateral data referred to earlier in the paper allowed for discussion and contextual consideration of emergent themes during the analytical process.

## 3 RESULTS

### 3.1 Participants

Fifty individuals were identified as eligible to participate from screening clinic lists. Of these, 23 (46%) participated in a semistructured interview (see Table 2 for demographic characteristics). Reasons for nonparticipation included: declined to participate (*n* = 15), unable to be contacted by the study team (*n* = 8), too unwell to participate (*n* = 10) and three returned consent forms after the recruitment period had ended due to substantial postal delays arising from the Covid‐19 pandemic. Audio‐recorded interviews lasted between 22 and 65 min (mean 49 min).

**Table 2:** Participant demographics.

Patients	Health service A (*n* = 15)	Health service B (*n* = 8)	Total (*n* = 23)
Age (years)			
Mean, SD	67, 8	67, 10	67, 8
Minimum, maximum	57, 82	53, 77	53, 82
Time since diagnosis (months)^a^			
Mean, SD	11, 7	15, 7	12, 7
Minimum, maximum	5, 31	4, 24	4, 31
Gender	*n* (%)^b^	*n* (%)^b^	*n* (%)^b^
Man	6 (40)	4 (50)	10 (43)
Woman	9 (60)	4 (50)	13 (57)
English first language			
Yes	9 (60)	4 (50)	13 (57)
No	6 (40)	4 (50)	10 (43)
Marital status			
Single	2 (13)	2 (25)	4 (17)
Married/de facto	6 (40)	4 (50)	10 (43)
Separated/divorced	6 (40)	1 (13)	7 (30)
Widowed	1 (7)	1 (13)	2 (9)
Aboriginal and/or Torres Strait Islander			
No	15 (100)	8 (100)	23 (100)
Current employment status			
Employed (full time/part time)	1 (7)	0 (0)	1 (4)
Not in paid employment	6 (40)	4 (50)	10 (43)
Retired	8 (53)	4 (50)	12 (53)
Highest level of education			
Partial secondary	10 (67)	7 (88)	17 (74)
Completed secondary (year 12)	2 (13)	0 (0)	2 (9)
Trade/TAFE	1 (7)	1 (13)	2 (9)
University	2 (13)	0 (0)	2 (9)
Diagnosis			
Lung cancer (did not specify)	10 (67)	5 (62)	12 (52)
NSCLC stage III/IV	5 (33)	3 (38)	11 (47)

Abbreviations: NSCLC, non‐small cell lung cancer; TAFE, Technical and Further Education.

a *N* = 22.

b Percentages may not total 100 due to rounding.

### 3.2 Qualitative analysis

Three key themes were identified through qualitative analysis, described in Table 3. The first theme describes the components of care participants described as being valuable in addressing their needs. The second theme provides insights into the benefits participants described of receiving valued aspects of care. The third and final theme reports the consequences of missed opportunities for valued components of care.

**Table 3:** Qualitative themes and subthemes.

Theme 1. Valued components of supportive care
Subtheme 1.1. Ongoing opportunities for consultation
Subtheme 1.2. Person‐centred information
Theme 2. Benefits of valued supportive care
Subtheme 2.1. Empowerment through tailored information
Subtheme 2.2. Autonomy: Improved confidence and strength
Subtheme 2.3. Reducing missed opportunities for care
Theme 3. Consequences of missed supportive care
Subtheme 3.1. Exclusion from participation in treatment and care decision‐making
Subtheme 3.2. Suboptimal care experiences
Subtheme 3.3. Feeling lost in the system
Subtheme 3.4. Feeling unsafe

#### 3.2.1 Valued components of supportive care

The valued components of care described by patients consistently reflect the established dimensions of cancer supportive care (information, social, functional, emotional, practical, psychological and spiritual support).^11^ These were evident across all participant narratives.

On the whole I was happy with the information I got because, about what was happening, as far as they knew. I'm not sure if anyone can prepare you for other things. You know how it affects you mentally, I'm not sure that, if you can be prepared. (PA18)

Opportunities for ongoing consultation regarding supportive care needs and provision of person‐centred information were commonly identified as valued components of care by participants.

##### Ongoing opportunities for consultation

Opportunity to discuss concerns with their treating team throughout their illness enabled patients to access the information and support they needed in a timely manner. However, the context in which these opportunities occurred varied across participants. Some described how each visit to the hospital began with a consultation with a nurse, followed by time with a doctor, ensuring that a breadth of issues was explored and discussed with different members of their team. Others described regular face‐to‐face or telehealth consultations with a specialist cancer nurse to follow up on, or identify new, concerns or needs. Several participants had accessed home‐based palliative or community care services, and received regular visits or telephone calls by team members to check in.

Generally, it's been a home visit, the last one was actually a phone call, but because everything's sort of bubbling along nicely they didn't bother following through with a visit and offered the 4 week in front of us type visit again. (PA12)

Participants particularly valued a formalised approach with regularly scheduled appointments with their health professional(s), as such consultations ensured regular contact with their treating teams and legitimised their concerns. Many participants who accessed ongoing opportunities for consultation conveyed an acute awareness of the busy, under‐resourced nature of health services. Many expressed that they were reluctant to ‘bother’ their treating team with concerns they considered trivial or inappropriate.

I say to them, ‘you've got far more important things to do, like there's people really ill that you should be visiting’ and they say, ‘no we beg to differ, you know, we need to be seeing you too’. But I feel like I don't want to waste their time, but on the other hand I do want to see them and just make sure I'm in touch. (PA8)

However, not all patients experienced the same opportunity to discuss their needs as a routine component of care. For some, these opportunities were informal and ad‐hoc and they consequently missed chances to discuss their needs and concerns. This was particularly pertinent for patients who interacted with their treating team over telehealth or those who received treatment at home (e.g., oral cancer therapies). Missing opportunities to routinely discuss concerns left some participants feeling disconnected and isolated from care.

I don't even feel like I've got a team to be honest. (PA3)

##### Person‐centred information

Receiving person‐centred information was another valued component of supportive care that empowered patients; enabling them to feel in control of their situation and aware of what services were available to them. This refers to receiving reliable and relevant information, at the right time, and in the participant's preferred format.

Having reliable, relevant, and personalised information encouraged participants to feel supported during their cancer journey. One participant described feeling connected with a wide range of contacts who were able to provide her with information to address any concerns.

It made it a lot easier to go into treatment knowing that there was so much there that I could access. There was a lot of information that I could get hold of. And even if the information I was given originally might've been a bit general I could always focus in on something that was specific to me and find and ask somebody, and there was always someone that could say, ‘yeah well this is who you go to, this is who you can speak to’. (PA4)

The provision of tailored information relevant to an individual's priorities at different times across their illness experience was key to feeling informed. This was contrasted to receiving lots of information all at once—particularly at diagnosis, when many participants described feeling emotionally overwhelmed and unable to absorb the information given to them.

You'd remember it better because it's something you want to know and how can it help you, you know. Because the system only works when you understand it and know what right questions to ask. It's not gonna work if you're just gonna get bombarded, you know what I mean, because you don't understand it, you can't comprehend anything. (PA20)

Preferences for information were highly individualistic, and participants discussed a broad range of sources and mediums by which they wanted to receive information. Some favoured talking in‐person with their treating team, while others preferred to seek out relevant information on their own.

I think you needed a person's name, not just ‘ring this number and we'll help you’, you needed some sort of contact, that emotional contact with another person and you knew who they were. (PB4)

#### 3.2.2 Benefits of valued supportive care

Participants described many benefits of receiving opportunities to discuss their needs and concerns and to receive tailored information. Benefits discussed included being empowered through tailored information, greater autonomy, improved confidence and strength, and reduced missed opportunities for care.

##### Empowerment through tailored information

Being given relevant information at the right time created a sense of safety for participants, and reduced the stress associated with the cancer and its treatments. Knowing how to access, or being linked in, with services, meant that patients were able to quickly address concerns and experienced greater peace of mind as a result. One participant articulated how this resulted in her feeing well supported during her cancer journey.

Well it relieves a lot of stress, you know, and doesn't make you feel as bad, you know, like things aren't as bad as what you think they are, because you've got the support, there are people you can talk to, you know. I've got a cancer support nurse, you get social workers there, you've got everything there that you need at any time. I've always got phone numbers and stuff that I could ring whenever I wanted to if I needed to talk to somebody. (PA6)

Participants who were happy with the information they received described feeling supported and valued by their treating team. This is illustrated by one participant who had received his diagnosis approximately 3 months before the interview. His treatment plan had not yet been finalised, however he reported being extremely happy with the amount of information given him to him about the process and what to expect. This had given him a sense of assurance that his treating team valued his health and allowed him to feel in control of his situation.

They were just right up front with where I'm going, what I'm doing, how are we going, this is what we're going to do, they were absolutely perfect. (PB1)

Many participants articulated ways in which opportunities for conversations helped them manage concerns. For some, knowing there was an opportunity for regular check‐ins gave them greater confidence in their ability to manage their symptoms and side effects at home. Knowing that they had trusted sources of information available for questions and concerns as they arose resulted in participants' feeling empowered. This was keenly illustrated by one participant who had access to a specialist nurse as a source of information. The participant had struggled with deeply upsetting side effects and found the advice provided by the nurse gave her strength to continue treatment.

I think I would've despaired; I might've even stopped quite early if it hadn't been for her. (PA18)

##### Autonomy: Improved confidence and strength

Participants described how opportunities to discuss concerns and appropriate information gave them autonomy over whether they accessed services or not. For some patients, not accessing services enabled them to retain a sense of control over their lives. Some people had a strong preference for managing their needs on their own, or with the assistance of supports such as family and friends or their general practitioner (GP), rather than relying on hospital services. Asking for assistance from these supports was far easier due to their pre‐existing relationship and by contrast, several participants voiced that they felt uncomfortable opening up to, or receiving assistance from, strangers employed at their health service. For others, eschewing services was a way to retain independence. One participant who had received a terminal diagnosis discussed how she was not yet ready to engage with services ranging from peer and financial support through to funeral planning, though recognised these would be required at later stages. While some participants reported declining supportive care services, there was overwhelming agreement that knowing what hospital services were available was important, and participants discussed how knowing that help was there if needed gave them a sense of security and safety.

It is a peace of mind knowing that you could ring, yeah, it's not a useless thing, it's a peace of mind. (PA19)

##### Reducing missed opportunities for care

Participants who were well connected with their team and provided with relevant, tailored information described instances where they consequently accessed services they did not realise existed, or that they were eligible for. A participant who had been referred for home care through her health service noted that she wouldn't have thought to ask hospital staff about such services.

You know, I wouldn't say ‘well can someone come and clean my shower’. (PA3)

Another participant, who had been on the public housing wait list for 4 years, recounted how she had been discussing her accommodation arrangements with a specialist nurse. Unbeknownst to the participant, she was now eligible for priority housing due to her diagnosis, and the nurse arranged the requisite paperwork to be expedited.

So what she's done is she's wrote a massive, big referral and a letter from her, and she's had the occupational therapist come through here and mark down what I need and what cannot be touched here because it's a rental, and that I deserve to be considered for emergency housing with them as I've already been with them for 4 years. (PA4)

Additionally, tailored information about the role and benefits of recommended services helped to improve uptake. This clearly illustrated by participants' discussing their hesitancy in accepting referrals for palliative care services. Two patients living in the terminal stages of illness relayed they had refused palliative care referrals as they considered this service purely for end‐of‐life care, and that engaging with such services would be tantamount to admitting defeat.

I feel if I do it, I'm just giving up, and I don't want to give up. (PB11)

People came to see me to tell me, let me know what I should be doing. I haven't had pastoral care yet because that's my choice. I am going to do that very, very shortly. (PB2)

Other participants echoed associating palliative care with end‐of‐life services. However, after the provision of adequate information of the support this service provided through discussions and written resources, they were satisfied to accept the referral.

We just got word of mouth through the hospital; got a few numbers we rang which was palliative care… We got a bit worried when we heard palliative care, but they do a lot of other things now I know. (PB3)

#### 3.2.3 Consequences of missed supportive care

Participants described many consequences that arose when they did not receive ongoing opportunities to discuss their needs or person‐centred information. These consequences included being excluded from treatment decision making, suboptimal experiences of care and mitigable adverse events and feeling unsafe. This impacted whether people experienced care as valuable and valuing.

One doctor said, ‘oh the cancer's gone’. Which means what, is it still there? is it still, you know. Nobody's told me at all. The more you ask the more they avoid it, ‘oh I'll have to check on the records’, ‘we'll have to have a look’ … get lost, you know. (PB)

##### Exclusion from participation in treatment and care decision‐making

A key consequence of the absence of ongoing consultations and personalised information participants described was feeling excluded from treatment decision making. Many discussed feeling frustrated and distressed when their diagnosis and treatment plan were inadequately explained, such as why chemotherapy wasn't suitable. This resulted in some participants feeling unsure if all treatment avenues had been thoroughly explored, particularly regarding clinical trials and novel therapies. Other participants described feeling as though they were on a merry‐go‐round of appointments with different specialists for unknown reasons, which left them feeling upset and agitated.

I said to him, ‘when are you going to start the treatment, I keep coming here every two to three weeks to see an oncologist and nothing is happening’. He lost his shit, and he turned around and he said to me, ‘we don't know what to treat you for’ and it's like oh wow. I started crying. (PB10)

I'm a bit distraught today, because I'm not understanding it, that's all I'm asking you, can someone explain it in laymen's terms to me and not doctor's terms. (PA4)

##### Suboptimal care experiences

Participants described instances where a lack of person‐centred information resulted in suboptimal experiences of care. Information regarding treatment was crucial to prepare people for potential side effects of treatment. A participant who was prescribed a targeted therapy shared how she had delayed starting her treatment due to fear which could have been addressed through appropriate information provision.

She handed it to me and said, ‘this is the tablet you'll be taking’, and then she disappeared, like there was no information at all and that was really horrible because I was afraid to take the tablet. No one had explained to me what to do or how to go. (PA8)

Another participant recounted being taken to hospital by ambulance three times in 1 month due to severe side effects of treatment. He had spent several months without receiving information regarding, or referrals to, supportive care services such as community palliative care, which were eventually organised after he had spent over 8 h in the emergency department waiting room.

As of right now we're doing okay, we've sort of, like we've got all the contacts we need… Yeah it was just hard earlier on that's all, the first 6 months were really hard. (PB3)

##### Feeling lost in the system

Several participants reported how the absence of opportunities to discuss their needs and appropriate information left them feeling ‘lost in the system’. While some participants felt confident seeking information and navigating supportive care services on their own, others described feeling lost regarding how to access services and consequently missing out on care. In some instances, participants explained that they hadn't been offered information regarding what supportive care services were available. Others had identified services they needed but did not know how to get in touch with them. One participant, eager to connect with palliative care and advanced care planning, had not been able to contact these services due to a lack of contact with her health service since her treatment had ended.

I didn't know about palliative care, and I think one of my doctors in Melbourne mentioned it and I said, ‘well let's talk about it’, but nothing's happened. (PA15)

I don't think [it] was really ever mentioned… if you need to see a psychologist or you need, somewhere along that line, get in touch with this number. I don't think that was offered to me. And then again I don't even know whether I would've used it if they had, because as I say at the time your thinking is rather different I think. (PB4)

##### Feeling unsafe

Lack of timely and empathetic communication from care providers resulted in people feeling unsafe, or perceiving care as lacking in value or devaluing, resulting in disengagement from care. This was clearly illustrated by one participant who had built a relationship with a previous oncologist, allowing her to feel comfortable sharing her needs and concerns. She described her shock of being removed from this usual oncologist's care with no warning or explanation and revealed she had not received care from a consistent oncologist since. Consequentially, she had substantially disconnected from specialist care, instead preferring to pay out‐of‐pocket for GP appointments to have her needs addressed.

I just go to my GP at the moment. Before, when I had the oncologist most of the time, the same one, I could ask her, I felt good to ask her. Not now. Not now, I'd just rather go to my own medical centre. (PB10)

## 4 DISCUSSION

Through a detailed investigation of what aspects of care patients valued—that is, what mattered to them as they tried to cope with the demands of the disease and treatment, our study has highlighted the importance of integrating supportive care as a fundamental enabler of value‐based cancer care. Descriptions of care offered by participants illustrate how delivering care informed by ‘what matters most’ to each patient (person‐centred care) is a central facet of value‐based care. In their commentary on value‐based and patient‐centred care,^15^ Tsang and Hicks conclude that without explicit attention to person‐centred care, there is a risk that what matters most to people will be overlooked in a value‐based care model. Our study has demonstrated that opportunity for ongoing consultations about supportive care needs and person‐centred information were highly valued and offer tangible targets for value‐based care metrics; timely, person‐centred, and safe care.^13^

Opportunities for ongoing consultation and person‐centred information resulted in positive experiences and outcomes of care enabling timely discussion and management of priority concerns, coordinated care and patient confidence in their treating team. These findings support data previously reported.^19^ Moreover, the information needs and preferences of people affected by cancer are highly variable and fluctuate over time.^20^ Implementation of care components valued by participants in our study may enable care that is personalised to individual needs, and supports people to engage in self‐management (as they are able and desire) through timely navigation to supportive care resources, when and as they need them.^20^ The importance of tailored information was exemplified by participant discussions of palliative care. While many initially viewed the service as synonymous with treatment cessation and death (congruent with substantial literature),^21^ education about the broad scope of palliative care, and potential personal benefits of engaging with the service was useful to ensure patients felt comfortable accepting a referral to palliative care, and ultimately influenced uptake. Early palliative care has been shown to improve patient quality of life and duration of survival.^22^, ^23^ This study demonstrates how tailoring information to each individual are key to support patient‐centred decision making to facilitate timely access to services that best meet their needs and values.^24^

When patients did not have the opportunity for ongoing consultations about their needs, or did not receive patient‐centred information, they talked about missed opportunities for care such as: exclusion from participating in treatment decisions due to inadequate information, inability to reach required services and disengagement from care. Participants also described avoidable adverse events such as unplanned presentations and distress due to not understanding care and treatment, when information was not tailored to their needs or information was not accessible at times when care needs changed. This impacted their feelings of safety and of being valued in the health system. Importantly, as described in other studies, participants explained that when they felt safe and valued, they felt better able to cope with the demands of their cancer and treatment.^25^, ^26^

While individual patient needs and preferences were diverse, participants strongly endorsed ongoing opportunities to have their needs recognised and addressed. Implementation of routine supportive care screening processes offers opportunities for timely identification of dynamic needs and reduced barriers to care, thereby improving outcomes that matter most to patients.^27^ Embedding systematic screening processes will enable all patients to have access to consultations, irrespective of location of care, type or duration of treatment. This is especially important for populations at higher risk of suboptimal outcomes, for example, regional and rural patients, people with mental health issues, those with low health literacy and whose preferred language is not English. These processes may be particularly important for people with lung cancer who are known to underutilise healthcare, despite the severe symptomatology of their disease.^7^, ^28^

Heterogeneity of needs, preferences, and outcomes within and across patient populations pose challenges to healthcare planning at a system level; however, our study demonstrates that being attentive to what is valued by and matters most to patients is crucial to achieving value‐based cancer care. Integrating standardised processes for supportive care needs screening and assessment, as a component of routine cancer care, will contribute to achieving value‐based care for all cancer patients.

### 4.1 Strengths and limitations

This study has limitations to note. Results report the perspectives of people with lung cancer attending two tertiary metropolitan health services, who may have different priority needs to those living in regional/remote locations. The experiences of individuals who are Aboriginal or Torres Strait Islander were not captured, and we recognise that their experiences of and priorities for care may be very different. Further research with populations at risk of poor health outcomes because of disease or social determinants will enhance evidence presented. However, 43% of participants did not speak English as a first language, and the majority had not completed secondary schooling, which reflects the demographic incidence of lung cancer. The centrality of opportunities for consultation and personalised information in shaping participants' experiences of care, despite their diversity of priorities, gives persuasive evidence that these are important components in helping lung cancer patients cope with the demands of their cancer and treatment.

## 5 CONCLUSION

Qualitative interviews with people with lung cancer have shown that the opportunity to discuss priority needs across the pathway of care, and provision of person‐centred information are valued components of cancer care. Accordingly, we recommend that integration of standardised supportive care screening and assessment and personalised information should be prioritised to achieve value‐based lung cancer care. When care overlooks what is valued by and matters most to people, perceptions of trust and safety in care are diminished, and patients experience suboptimal health outcomes.

## AUTHOR CONTRIBUTIONS

**Holly Chung**: Investigation; formal analysis; writing—original draft; writing—review and editing. **Amelia Hyatt**: Writing—review and editing; project administration; formal analysis; writing—original draft; methodology. **Kate Webber**: Writing—review and editing. **Suzanne Kosmider**: Writing—review and editing. **Meinir Krishnasamy**: Conceptualisation; methodology; supervision; writing—original draft; writing—review and editing; funding acquisition.

## CONFLICT OF INTEREST STATEMENT

The authors declare no conflict of interest.

## ETHICS STATEMENT

This project was reviewed and approved by Peter MacCallum Cancer Centre Human Research Ethics Committee (HREC) (multisite approval number: HREC/66771/PMCC). All study participants gave informed written consent after the purpose of the study and their involvement was explained to them. All transcripts were deidentified, and participants were referred to by code numbers rather than names to ensure confidentiality. The methods employed by this study are in accordance with the principles and standards in the Declaration of Helsinki.

## Data Availability

The data that support the findings of this study are available from the corresponding author upon reasonable request.
